# Diclofenac-Loaded Orodispersible Nanofibers Prepared by Double-Needle Electrospinning

**DOI:** 10.3390/polym17091262

**Published:** 2025-05-06

**Authors:** Luca Éva Uhljar, Tekla Jáger, Csongor Hajdu, Anett Motzwickler-Németh, Orsolya Jójárt-Laczkovich, Martin Cseh, Katalin Burian, Rita Ambrus

**Affiliations:** 1 Institute of Pharmaceutical Technology and Regulatory Affairs, Faculty of Pharmacy, University of Szeged, Eötvös Street 6, H-6720 Szeged, Hungaryjagertekla@gmail.com (T.J.); csongi982010@gmail.com (C.H.); nemeth.anett@szte.hu (A.M.-N.); jojartne.laczkovich.orsolya@szte.hu (O.J.-L.); 2 3D Center, Center of Excellence for Interdisciplinary Research, Development and Innovation, University of Szeged, Tisza Lajos Blvd. 107, H-6725 Szeged, Hungary; cseh.martin@szte.hu; 3 Department of Medical Microbiology and Immunobiology, University of Szeged, Dóm Square 10, H-6720 Szeged, Hungary; burian.katalin@med.u-szeged.hu

**Keywords:** diclofenac, double-needle electrospinning, drug delivery system, electrospinning, fast-dissolving, nanofiber, NSAID, orodispersible

## Abstract

The main aim of this study was to develop a diclofenac-loaded, orodispersible formulation prepared by double-needle electrospinning. For the use of two needles, one above the other, a new needle holder was designed and 3D printed. During the optimization of the drug-free PVP carrier, the effect of the polymer concentration on the morphology and average fiber diameter was investigated. Electrospinning was possible for solutions with a PVP concentration between 7.5 and 15 *w*/*w*%. Too low viscosity led to smooth-surfaced nanoparticles, since electrospraying occurred. The optimal material properties and process parameters were used to prepare drug-loaded nanofibers. The morphology, crystallinity, chemical interactions, encapsulation efficiency, drug distribution, in vitro disintegration, in vitro dissolution, cytocompatibility, and 6-month stability were tested. According to the results, the electrospun formulation was an amorphous solid dispersion with excellent encapsulation efficiency. The drug distribution was homogeneous within the nanofiber matrix. The disintegration was completed in about 5 s in artificial saliva and about 41 s on an artificial tongue. The dissolution in artificial saliva was complete within 10 min. Overall, a promising formulation was developed with rapid disintegration, immediate drug release, and good stability. Additionally, a new in vitro dissolution method (“AS-to-FaSSGF”) was developed to obtain a bigger picture of drug dissolution throughout the gastrointestinal tract.

## 1. Introduction

Over the past decades, the demand for orally dispersible formulations has increased significantly due to their convenience and improved patient adherence [1]. Orodisperse systems rapidly disintegrate upon contact with the tongue. They can be easily administered without the need for water, making them ideal for situations with a lack of liquid (e.g., traveling) [2]. Also, orally disintegrating formulations offer a significant advantage for patients with swallowing difficulties, e.g., pediatric, elderly, and hospitalized patients, as well as individuals experiencing nausea, vomiting, or motion sickness [3]. According to the European Pharmacopoeia, an orodispersible dosage form is defined as having a disintegration time of less than 3 min [4]. In general, the disintegration time of dispersible formulations ranges from a few seconds to approximately one minute [5].

Nanofibrous matrices and films can be good candidates as orodispersible formulations due to their small fiber diameter, large specific surface area, and connected porosity [6]. Also, a wide variety of polymers can be electrospun, and the use of a hydrophilic polymer as a fiber-forming matrix also facilitates the formulation of fast-dissolving drug delivery systems [7]. Commonly used polymers are the polyvinylpyrrolidone (PVP) [8], polyvinyl alcohol (PVA) [9], hydroxypropyl methylcelluloses (HPMC) [10], polyethylene oxide (PEO) [11], gelatin [12], hydroxypropyl-beta-cyclodextrin (HP-βCD) [13], or combinations of these [14].

Electrospinning (ES) is the most widely used method for nanofiber (NF) production. It is an electrohydrodynamic process in which NFs are drawn from an ES solution under the influence of high voltage. The principle behind the process is that a strong electric field induces the ejection of a thin liquid jet from a droplet. As the jet travels toward the collector, it undergoes elongation, stretching, and solidification, forming NFs. The ES process and the produced NF morphology are influenced by several factors which can be grouped as material properties, process parameters, and ambient parameters [15]. Among the material properties, every aspect that affects the viscosity and conductivity of the ES solution is important. The most important properties are the polymer concentration, and the type of the ingredients. Among the process parameters, the key factors are the type and number of the spinneret, and the applied high voltage. The traditional ES contains one needle as a spinneret. This single-needle ES is the most used method in academic research; however, industrial-scale production requires multiple needles connected side by side [16]. The main reason for the need for the multi-needle ES is the low productivity of the single-needle method (capacity of ∼0.01–0.1 g/h) [17]. However, the multi-needle ES is challenging because the closely flying jets interact and modify the electric field, making NF collection more difficult since it can lead to the formation of multiple fiber deposition areas on the collector, rather than a single, uniform patch [18].

This study aimed to produce and investigate a diclofenac-containing orodispersible formulation prepared by double-needle ES. To the best knowledge of the authors’, this is the first reported double-needle ES method for diclofenac-loaded NFs. After optimization of the preparation method, the formulation was extensively tested to determine its suitability for the development of an orodispersible dosage form. As part of the investigation, a novel in vitro dissolution method, the so-called “AS-to-FaSSGF” method, was developed.

## 2. Materials and Methods

### 2.1. Materials

Diclofenac sodium (DS; Mw = 318.13) was donated by PannonPharma Llc. (Pécsvárad, Hungary) for research work. Polyvinylpyrrolidone (PVP; Mw = 1,300,000) and ethanol (96% purity) were purchased from Thermo Fisher Scientific Inc. (Budapest, Hungary).

Phosphate-buffer solution (PBS; pH 7.4) was prepared in-house and used as a solvent for the drug loading (DL%) and entrapment efficiency (EE%) measurements. For the in vitro disintegration and dissolution studies, different artificial secretions, namely artificial saliva (AS) and Fasted State Simulating Gastric Fluid (FaSSGF), were prepared in-house. The recipe of the AS solution was as follows; measures of 8 g sodium chloride, 2.38 g disodium hydrogen phosphate, and 0.19 g potassium dihydrogen phosphate (all purchased from Molar Chemicals Ltd., Halásztelek, Hungary) were dissolved in 1 L distilled water, and the pH was adjusted to 6.8 by the addition of phosphoric acid. The FaSSGF medium was prepared according to the instructions of the producer, Biorelevant.com Ltd. (London, UK). HPLC-grade methanol (Promochem LGC Standards GmbH., Wesel, Germany) and PBS (pH 2.5) were used as eluents during the separation and detection of the dissolved drug.

For the in vitro cytotoxicity study, Minimal Essential Medium (MEM) with Earle’s salts (Merck KGaA, Darmstadt, Germany) and MTT solution (thiazolyl blue tetrazolium bromide; Merck KGaA, Darmstadt, Germany) were used.

All the chemicals, except the HPLC-grade methanol, were analytical grade, and purified water was used.

### 2.2. Preparation of the ES Solutions

The first step was to optimize the fabrication of drug-free PVP NFs. Five ES solutions with different PVP concentrations (5, 7.5, 10, 12.5, 15%) were prepared by dissolving the required amounts of PVP (Mw = 1,300,000) in ethanol. After overnight magnetic stirring, the viscosity of the ES solutions was measured using an IKA Rotavisc me-vi rotating viscosimeter (IKA-Werke GmbH & Co., Staufen, Germany), equipped with a type SP-11 spindle.

After finding the best polymer concentration, the drug was loaded into the NFs. The required amounts of PVP and DS were dissolved in ethanol and stirred overnight to obtain the ES solution of 10 *w*/*w*%. Additionally, the viscosity was measured as written above.

### 2.3. Nanofiber Preparation via Double-Needle Electrospinning

**Figure 1:**
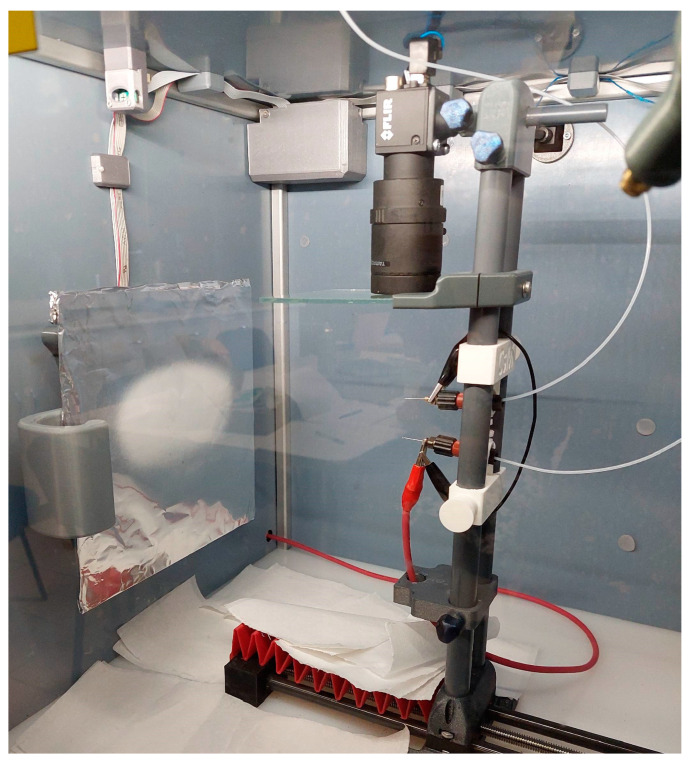
Electrospinning of PVP-based nanofibers using the double-needle method.

The NF preparation was carried out with the Spincube electrospinning machine (Spinsplit Llc., Budapest, Hungary) (Figure 1). The ES solution was filled into the 5 mL syringes and fed into the 22-gauge needles at a constant rate. As a collector, a static metal plate coated with aluminum foil was used. The needle–collector distance was 17 cm, the chamber temperature was 24–25 °C, and the relative humidity was between 36 and 44%. The voltage was set between 10 and 16 kV during the optimization process.

### 2.4. Design and Preparation of the 3D-Printed Needle Holder

**Figure 2:**
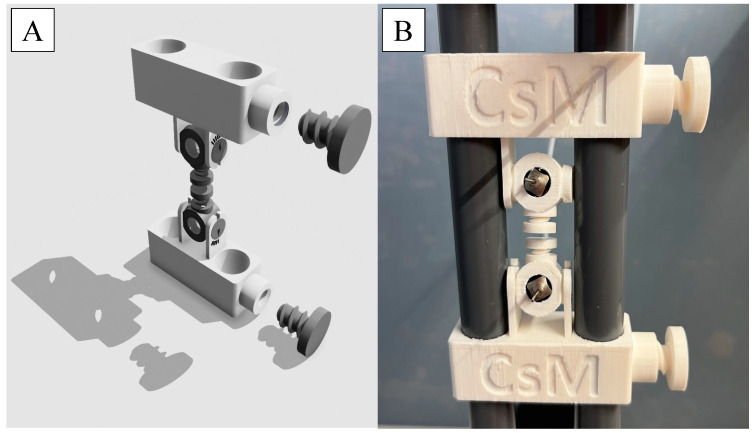
Design (**A**) and photo (**B**) of the 3D-printed needle holder for double-needle electrospinning.

One novelty of this research was the use of the double-needle ES setup. With the installation of the second nozzle into the instrument, the production rate could be doubled, or in other words, the preparation time would be halved. This required a new nozzle holder, which was designed using Shapr3D CAD software (version: 5.370; Shapr3D Zrt., Budapest, Hungary), as Figure 2A. shows. The design was exported in high-resolution .stl format and was sliced with Craftware software (version: 1.23; CraftUnique, Budapest, Hungary). The assembly was printed part-by-part with a CraftBot Plus Pro (CraftUnique, Budapest, Hungary) 3D printer with the following parameters: 0.2 mm layer height, along 15% parallel line infill with a 0.4 mm nozzle diameter and 60 mm/s draw speed. The PLA filament used was purchased from Herz Hungária Ltd. (Budapest, Hungary).

### 2.5. Preparation of the Physical Mixture

As a reference for the XRPD, Raman Spectroscopy, and in vitro dissolution studies, untreated DS powder and physical mixtures were used. The composition of the mixture was the same as the NF, namely 10 *w*/*w*% DS and 90 *w*/*w*% PVP. The homogenization was carried out in a shaker mixer (Turbula System Schatz; Willy A. Bachofen AG Maschinenfabrik, Basel, Switzerland) under controlled conditions (50 rpm, 10 min).

### 2.6. Physicochemical Characterization of the Nanofibers

The morphology of the electrospun fibers was examined by scanning electron microscopy (SEM; Hitachi S4700, Hitachi Scientific Ltd., Tokyo, Japan). The samples were coated with a gold–palladium coating (Bio-Rad Sputter Coater 502, VG Microtech, Uckfield, UK) before capturing. The fiber diameter and fiber diameter distribution were determined from the SEM images using ImageJ 1.53a software (U. S. National Institutes of Health, Bethesda, MD, USA).

The crystallinity was investigated by X-ray powder diffraction (XRPD; BRUKER D8 Advance Diffractometer, BRUKER AXS GmbH, Karlsruhe, Germany). The fibrous samples were scanned at 40 kV and 40 mA over an angular range of 3–40°. Kα2 radiation was removed from the diffractogram, and background correction, smoothing, and evaluation were performed using DIFFRAC plus EVA software (version 5.2; Karlsruhe, Germany). The physical mixture, untreated polymer, and untreated drug were used as reference powders.

The presence of components was evaluated using Raman Spectroscopy (Thermo Fisher DXR Dispersive Raman microscope, Thermo Fisher Scientific, Waltham, MA, USA) equipped with a CCD camera and a diode laser operating at a wavelength of 780 nm. Raman spectra were recorded in the spectral range of 800–1900 cm^−1^, with an exposure time of 6 s. The same references were used as in the XRPD measurement.

The drug loading (DL%) and entrapment efficiency (EE%) of the NFs were calculated from absorbance. First, the NF samples were cut into 1 cm^2^ pieces at random locations and weighted using a calibrated high-accuracy balance (Mettler-Toledo AX205 DeltaRange analytical balance; Mettler-Toledo International Inc., Columbus, OH, USA). Afterwards, the specimens were perfectly dissolved in 10 mL of pH = 7.4 PBS. Finally, the solutions were measured using a UV-Vis spectrophotometer (ABL&E-Jasco UV/VIS Spectrophotometer V-730, Budapest, Hungary) at 277 nm. The DS concentration was calculated using a calibration curve (y = 27.588x, R^2^ = 0.9985). Measurements were performed six times, and the mean and standard deviations were reported.

The drug distribution within the NF matrix was studied using Raman Chemical Mapping (Thermo Fisher DXR Dispersive Raman microscope, Thermo Fisher Scientific, Waltham, MA, USA). Specimens of 1 cm^2^ were randomly cut out from the NF matrix, and Raman spectra were collected from an 800 × 250 µm area with 30 measuring points in the spectral range of 3300–200 cm^−1^. The exposure time was 6 s and an average of 12 scans was applied. Determining the distribution of DS in the specimens, the C-C double bond at 1600 cm^−1^ was selected for profiling.

### 2.7. In Vitro Disintegration Tests

Two methods were used to investigate the disintegration behavior of the NF samples. Firstly, free immersion into 25 mL of AS was executed in Petri dishes. Secondly, a more developed method was used, in which the physiological conditions of the surface of a moist tongue were simulated. This method was first reported by Bi et al. in 1996 [19], and is nowadays commonly used for the investigation of fast-disintegrating NFs [6,13,20]. In our test, the NF samples were examined with a slightly modified version of this technique. A filter paper with 7 cm diameter was soaked with AS, drained of excess moisture, and placed in a dry Petri dish. Then, a 2 cm^2^ specimen of the NF mat was placed onto the wet paper. In both methods, the tests were performed in triplicate, the disintegration was recorded by a digital camera at 30 fps, and the disintegration time was calculated.

### 2.8. In Vitro Dissolution Tests

Two types of in vitro dissolution studies were executed to obtain a complete picture of the DS release from the NFs. Firstly, AS, as a single medium, was used in a thermostatic beaker with real-time UV probe detection (AvaLight DH-S-BAL spectrophotometer connected to an AvaSpec-2048L transmission immersion probe; AVANTES, Apeldoorn, The Netherlands). A measure of 2.5 mg untreated DS powder, 25 mg NF (containing 2.5 mg DS), and 25 mg physical mixture (containing also 2.5 mg DS) were placed into 200 mL AS and stirred with 200 rpm (DLAB MS-H280-Pro magnetic stirrer; DLAB Scientific Co., Ltd., Beijing, China) at 37 °C for 10 min. The UV absorbance was measured in real time at a 277 nm wavelength, but the averages calculated from the following points were plotted on the graph: 0.17, 0.33, 0.50, 0.67, 0.83, 1.00, 2.01, 3.01, 4.02, 5.02, 6.03, 7.03, 8.04, 9.04, 10.03 min.

In the case of orodispersible formulations, the disintegration and the dissolution mainly take place in the oral cavity. However, after disintegration, the saliva mixed with the medicine can be swallowed. This allows the drug to enter the stomach and meet with the gastric juice. Since testing of this idea is currently not found in the literature on orodispersible formulations, a new method for modeling this natural process had to be developed. So, a new “AS-to-FaSSGF” in vitro drug release method was developed as follows. The samples were placed into 5 mL of AS and gently shaken. After exactly one minute, the liquid was poured into the tank of a paddle-type dissolution apparatus (Hanson Research SR8-Plus Dissolution Test Station, Chatsworth, CA, USA) already containing 895 mL of FaSSGF medium. The paddles rotated at a speed of was 200 rpm, and the medium was 37 °C. The sampling points were at 1, 5, 10, 15, 30, 45, 60, and 90 min. At each sampling point, 1 mL aliquot was withdrawn, filtered, and measured by HPLC.

High-performance liquid chromatography (HPLC) was used to determine the dissolved DS concentration in the AS-to-FaSSGF method. Chromatographic separation was performed by an Agilent 1260 HPLC (Agilent Technologies, Santa Clara, CA, USA) using a Kromasil^®^ C18 (4.6 × 150 mm, 5 µm; Nouryon, Bohus, Sweden) analytical column at 40 °C. The mobile phase was a 1:3 mixture of pH 2.5 PBS and methanol. The flow rate of the isocratic elution was 1 mL/min. The DS concentration was determined at 273 nm using a UV-Vis diode array detector. The retention time of the drug was 2.76 min. Data were evaluated using ChemStation B.04.03. Software (Agilent Technologies, Santa Clara, CA, USA).

To determine the kinetics of the drug dissolution, five different mathematical models (zero order, first order, Hixson–Crowell, Higuchi, and Korsmeyer–Peppas) were fitted to the in vitro dissolution curves. The model with the highest regression coefficient (R^2^) was selected as the best at describing the kinetics.

### 2.9. Biocompatibility Studies

MTT assay was performed to evaluate the effect of the NF samples on cell viability. Caco-2 human colon carcinoma cell line (ATCC, Manassas, VA, USA) was cultured in 96-well plates at a seeding density of 4 × 10^4^ cells per well. A measure of 100 µL MEM with Earle’s salts medium was used, supplemented with 20% heat-inactivated fetal bovine serum (FBS), 2 mmol/L L-glutamine, non-essential amino acids, MEM vitamins, sodium pyruvate, and nystatin. Cells were incubated for 24 h at 37 °C in a humidified atmosphere containing 5% CO_2_.

After the first incubation, the medium was aspirated, and the cells were treated with the test samples. All conditions were assessed in triplicate. The initial concentration of the DS was 1 mg/mL, and two-fold serial dilution was used. So, the final 1:512 dilution resulted in a 1.953 µg/mL DS concentration. Untreated cells served as the positive control, while MEM without samples was the negative control. The plates were incubated for a further 24 h at 37 °C.

After the second incubation, 20 µL of MTT was added to each well, and the plates were incubated for an additional 4 h. Finally, 100 µL of 10% SDS in 0.01 M HCl was added to each well, and the plates were incubated overnight.

Cell viability was assessed by measuring the optical density at 570 nm, with a reference wavelength of 650 nm, using an EZ READ 400 ELISA reader (Biochrom, Cambridge, UK). Viability was expressed as a percentage of the control cells, calculated according to previously described methods [21]. Statistical analyses were performed using GraphPad Prism 8.0.1 (GraphPad Software Inc., San Diego, CA, USA).

### 2.10. Stability Studies

The storage stability of the DS-loaded NFs was investigated after 6 months of storage in a desiccator. The fresh and the stored samples were investigated in three aspects. The morphology was analyzed using SEM, the crystallinity was checked by XRPD, and chemical stability was tested using Raman Spectroscopy. All methods are described above.

### 2.11. Statistical Analysis

Statistical analysis was conducted to evaluate significant differences in the measured data. A one-way analysis of variance (ANOVA), followed by a post hoc Tukey HSD test, was performed to compare the in vitro dissolution data. The dissolved DS amounts from the DS-PVP NF, the DS-PVP physical mixture, and the pure DS powder were compared in all time points. The results with *p* < 0.05 were assumed to be statistically significant.

## 3. Results

### 3.1. Morphology of the Drug-Free PVP Nanofibers

In the case of drug delivery systems, it is advisable first to develop the carrier system and subsequently load it with the active ingredient. This principle also applies to NFs. As a first step, the optimization of drug-free PVP NF production was carried out. The preparation of NFs is influenced by numerous parameters, among which the most critical are the viscosity of the precursor solution and the applied voltage. The viscosity is mainly determined by the molecular weight and concentration of the polymer. A sufficiently high viscosity enables the formation of uniform, homogenous, and smooth-surfaced NFs. In contrast, low viscosity results in bead-like structures or leads to dripping and/or electrospraying instead of electrospinning. On the other hand, the relationship between applied voltage and fiber diameter in electrospinning is complex and can vary depending on specific experimental conditions. While some studies suggest that increasing the applied voltage leads to thinner fibers due to greater stretching forces [22], others report that higher voltages can result in thicker fibers or bead formation due to jet instability [2]. The effect of increased voltage on the fiber diameter may depend on factors such as polymer concentration, solution viscosity, and ambient conditions.

In this study, the effect of the viscosity of the ES solution on the fiber diameter and morphology was studied. The voltage was set between 10 and 15 kV in an effort to yield the optimal preparation in each case.

**Figure 3:**
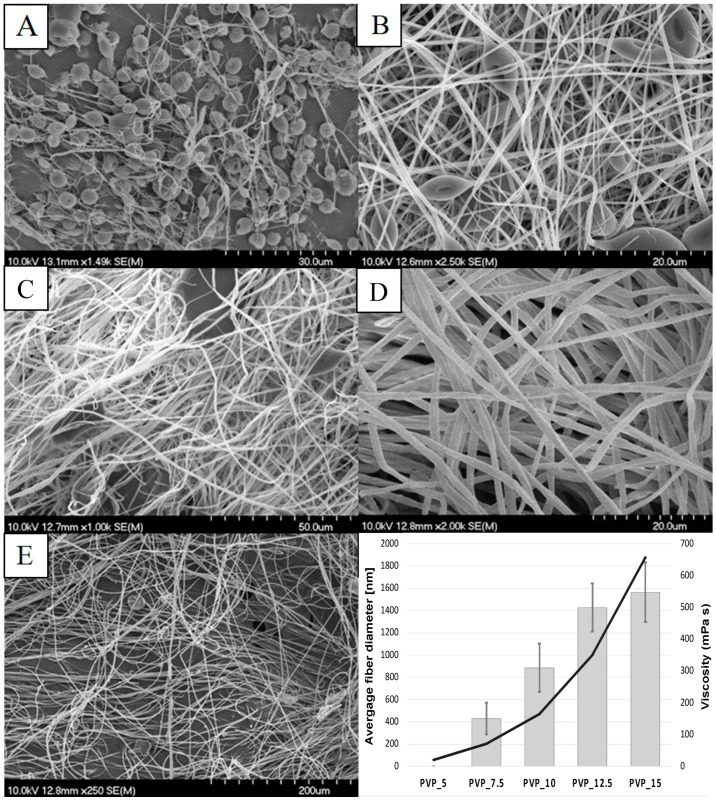
SEM images of the drug-free PVP nano- and microfibers with a graph showing the relationship between viscosity and fiber diameter. (**A**) PVP_5; (**B**) PVP_7.5; (**C**) PVP_10; (**D**) PVP_12.5; (**E**) PVP_15.

Five different ES solutions (5, 7.5, 10, 12.5, 15 *w*/*w*%) were prepared by dissolving the PVP in ethanol. Then, the viscosity was measured, and the solutions were electrospun. As was expected, the viscosity of the ES solutions increased with the increase in the polymer concentration (Table 1). A similar positive correlation between viscosity and average fiber diameter was found. The diameter of the drug-free formulations increased as the ES solution became more concentrated. A steady increase was found between 5 and 12.5 *w*/*w*%. However, the difference between 12.5 and 15 *w*/*w*% was not significant (Figure 3).

The SEM images showed that the 5 *w*/*w*% PVP concentration was below the limit for the fiber formation, resulting in electrospraying (Figure 3). Some fibers were present, but mostly spherical aggregates formed during the process. From the 7.5 *w*/*w*% PVP solution, red-blood-cell-shaped structures were observed, indicating insufficient viscosity. In the case of the 10 *w*/*w*% sample, beads and aggregates were observed occasionally but were still present. Additionally, the average fiber diameter exceeded 1 micrometer, so it is more appropriate to refer to the sample as microfibers rather than NFs. The morphology of the 12.5 *w*/*w*% fibers was good, with no beads, a uniform fiber diameter, and a smooth surface. The average fiber diameter was 1.427 ± 0.216 μm, classifying the fibers as microfibers. Finally, the 15 *w*/*w*% polymer solution was too concentrated, resulting in fibers with a ragged surface and an average diameter exceeding 1.564 ± 0.267 μm.

In conclusion, the viscosity of the ES solution significantly influenced the ES process. Fiber formation was inadequate at concentrations below 10% and above 12.5%. The optimal morphology of the drug carrier system was achieved using a 12.5 *w*/*w*% PVP solution filled in two syringes, connected to two 22 G needles. The voltage applied to the double-needle ES equipment was 16.5 kV with a 0.5 mL/h feeding rate.

### 3.2. Morphology of the DS-Loaded Nanofibers

The next step was the incorporation of DS into the microfibers. The aim was to achieve 10 *w*/*w*% drug content in the dry formulation. A content of 12.5 *w*/*w*% PVP and a sufficient amount of DS were perfectly dissolved in ethanol. The viscosity of the ES solution was 371 mPa·s, which was close to the drug-free solution (350 mPa·s). So, the addition of the DS did not result in a significant change in the viscosity. Therefore, the same process parameters were used during the double-needle ES.

The double-needle ES was successful as a relatively thick (0.53 ± 0.12 mm) and flexible fibrous membrane was collected. The handling of the membrane was convenient since it was easily removable from the aluminum foil and foldable to the desired size. The manufacturing of the NFs can be a challenge, so it is presumed that the very high molecular weight of the PVP was the reason for the flexible and adherent NF membrane.

**Figure 4:**
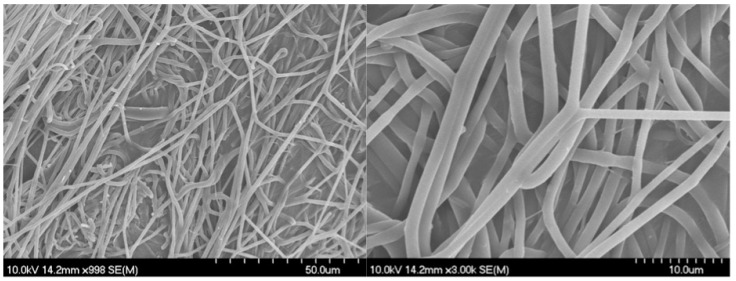
SEM images of the diclofenac sodium-loaded PVP nanofibers prepared by double-needle electrospinning.

The morphology of the DS-PVP fibers was appropriate, with long, bead-free, and smooth fibers (Figure 4). The fiber diameter was 896 ± 286 nm, which means a drastic decrease compared to the drug-free carrier (1427 ± 216 nm). Our hypothesis is that the addition of DS, being a salt, enhanced the conductivity of the ES solution. The increased charge in the jet led to greater elongation, resulting in the formation of thinner fibers.

### 3.3. Physicochemical Properties

Physicochemical properties of the electrospun formulation such as crystallinity, chemical bonding between components, drug content, and drug distribution were studied.

**Figure 5:**
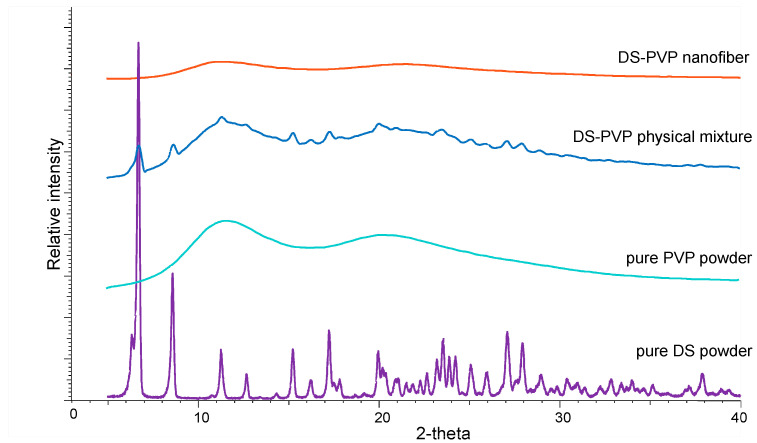
XRDP diffractograms of the crystalline diclofenac sodium (DS) powder and physical mixture and the amorphous PVP powder and DS-PVP nanofibers.

XRDP measurements confirmed the change in the crystallinity of the DS in the NFs. The characteristic peaks (6.5, 8.5, 11.2, 15.1, 16.2, 17.1, 20.0, 23.5, 25.1, 25.9, 27.1, 27.8, 37.8 2-theta) of the DS appeared in the diffractogram of the pure DS powder and the physical mixture but were completely absent from the fibrous sample (Figure 5). This proved that the NF sample was an amorphous solid dispersion where the DS was molecularly dispersed in the polymer.

**Figure 6:**
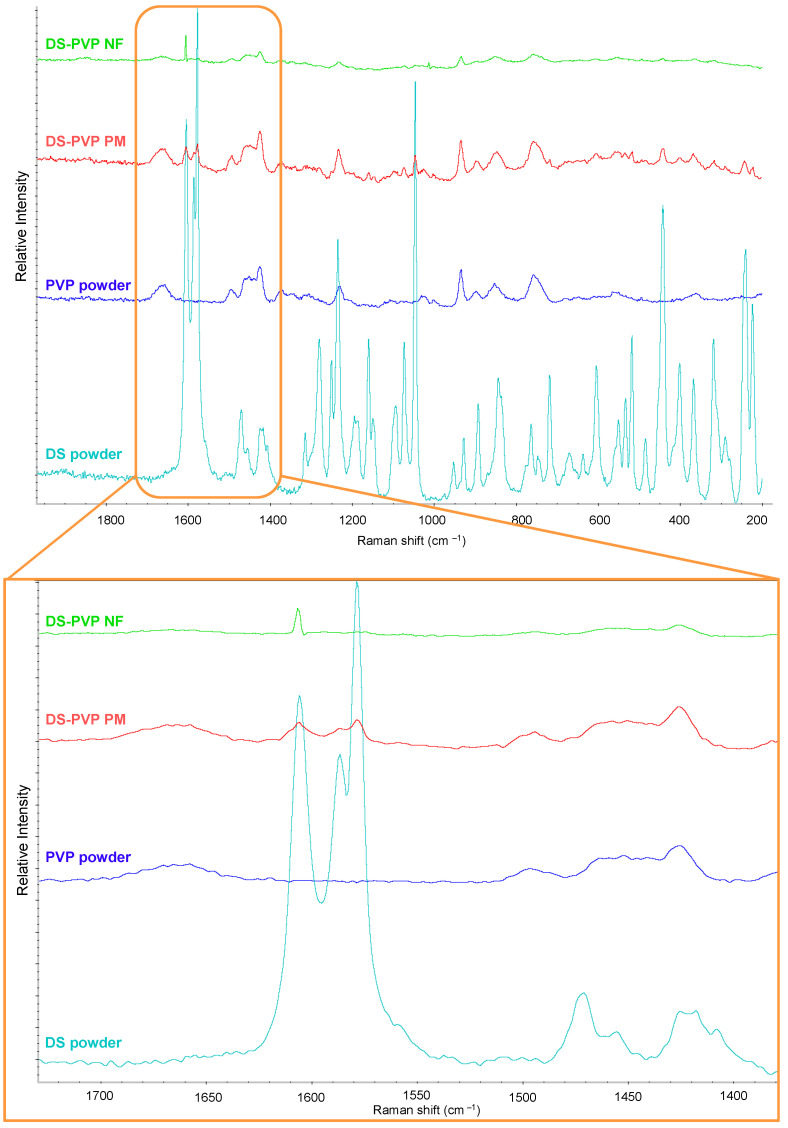
Raman spectra of the diclofenac sodium (DS), PVP powder, physical mixture, and the DS-PVP nanofiber.

The chemical structure and the presence of the DS were analyzed using Raman Spectroscopy. The characteristic fingerprint patterns of the pure DS and PVP powders were observed (Figure 6). In the case of the DS, the most intense, well-defined peak was a triplet around 1600 cm^−1^, containing the ring stretching vibrations of the phenylacetate at 1606 cm^−1^ and the dichlorophenyl ring at 1586 cm^−1^, and the asymmetric stretching vibration of the carboxylate at 1579 cm^−1^. This triplet is characteristic of the DS [23]. In the case of pure PVP, the fingerprint pattern appeared between 1510 and 1410 cm^−1^. Also, the characteristic pattern of both the drug and the polymer were distinctly visible in the spectrum of the physical mixture. On the spectrum of the NF, the characteristic peaks were also visible but appeared in lower intensity, indicating secondary interactions (e.g., hydrogen bond), and the formation of an amorphous solid dispersion.

The DL% and EE% were monitored by UV absorbance. The measured DS content was 9.96 ± 0.26%, which means 99.57 ± 2.59% entrapment efficiency. Very high EE% is usual in the case of NFs, since this preparation method is basically a rapid solvent evaporation.

**Figure 7:**
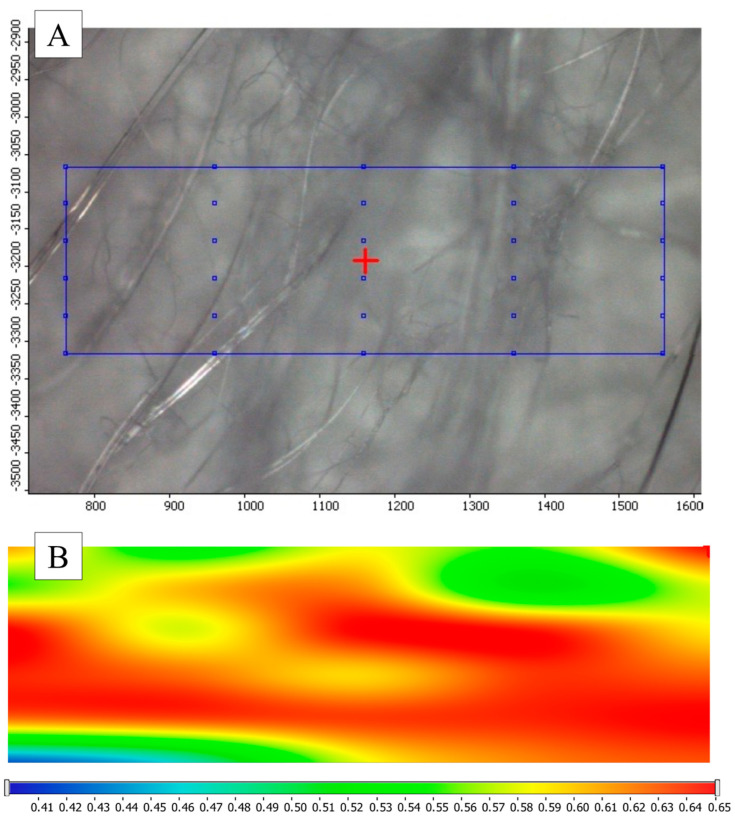
Microscopic image (**A**) and chemical mapping (**B**) of the diclofenac-loaded nanofibers.

The drug distribution within the NF sample was analyzed using Raman mapping. Figure 7 shows the microscopic image displaying 30 measurement points alongside the corresponding chemical map. In the chemical map, warmer colors indicate a higher concentration of the DS. The balanced coloration of the map suggests that the drug is homogeneously distributed throughout the NF matrix.

### 3.4. Rapid Disintegration Proved by Two Different Methods

Rapid disintegration is a key characteristic of orodispersible formulations. In this study, two different in vitro methods were used to determine the disintegration time of the DS-PVP NF matrix.

**Figure 8:**
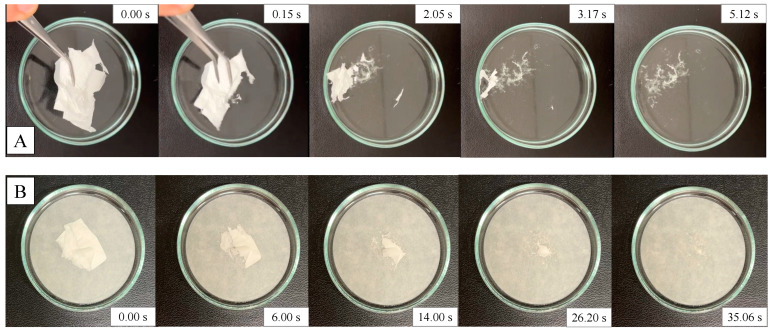
Two in vitro methods for the disintegration test of orodispersible nanofiber matrix loaded with diclofenac sodium. The processes of the free immersion method (**A**) and the artificial tongue method (**B**) are presented in five sequential frames.

First, the sample was immersed in 25 mL of AS (Figure 8A). Immediate disintegration was observed, with most of the formulation dissolving into the medium simultaneously upon wetting. The entire process was completed in 5.48 ± 1.53 s.

For orally disintegrating tablets, the U.S. Food and Drug Administration (FDA) requires a 30 s disintegration time using the United States Pharmacopeia (USP) method [24]. This method employs a constantly moving basket-rack assembly to test six tablets in one liter of distilled water. In contrast, our study used a smaller volume of AS and a stationary Petri dish for testing. Therefore, the disintegration time of the NF was notably fast.

The second in vitro disintegration test used wet filter paper to mimic the surface of the tongue. The same process was observed as the NF simultaneously wetted and disintegrated or dissolved on the surface of the filter paper (Figure 8B). The disintegration time was 41.38 ± 9.32 s. This result meets the requirements of a fast-disintegrating formulation.

Rapid disintegration is a critical parameter for an orodispersible NF matrix, as it facilitates the immediate release of the drug, making it readily available for dissolution and absorption.

### 3.5. In Vitro Dissolution in Artificial Saliva

**Figure 9:**
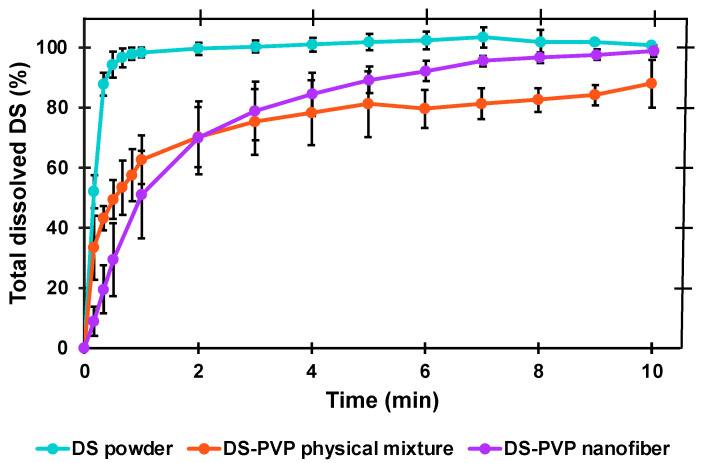
Dissolution in artificial saliva in the case of pure diclofenac sodium (DS), DS-PVP physical mixture, and 10 *w*/*w*% DS-PVP nanofiber matrix.

The in vitro dissolution of the drug from the NF formulation was tested by two methods. First, AS, as a single medium, was used, and the drug release from the NFs was compared with the dissolution of the pure DS powder and DS-PVP physical mixture (Figure 9). The pure DS powder showed rapid and complete dissolution in the pH 6.8 medium; since the solubility of DS is pH-dependent, the drug is highly soluble in alkaline solutions [25].

The presence of the PVP slightly prolonged the drug release by forming a viscous matrix, which slowed the diffusion of the DS. It was also confirmed by the kinetic models since the best-fitting mathematical model was the first order kinetics (R^2^ = 0.9520) in the case of the DS powder. This met our expectations as the drug dissolution in this case is not influenced by any external factors, and the process is driven by the concentration gradient. On the other hand, the dissolution kinetics of the DS-PVP physical mixture followed the Korsmeyer–Peppas model (R^2^ = 0.9464), in which both diffusion and polymer–drug interactions contribute to the overall release mechanism.

The data between the physical mixture and the NFs did not differ significantly until the fourth minute. From the fourth minute, the dissolution from the NFs was significantly higher (*p* < 0.05), and it even reached the curve of the pure DS at the last time point. Also, the shape of the curve was exponential, and the best-fitting dissolution kinetics was the first-order release (R^2^ = 0.9917). The released drug amount reached 80% within 4 min and the complete dissolution was achieved within 10 min. The FDA dissolution criterion for immediate release is Q = 80% in 30 min [26]. Thus, the tested DS-PVP NF can be considered an immediate-release formulation.

### 3.6. In Vitro Dissolution Tested by “AS-to-FaSSGF” Method

**Figure 10:**
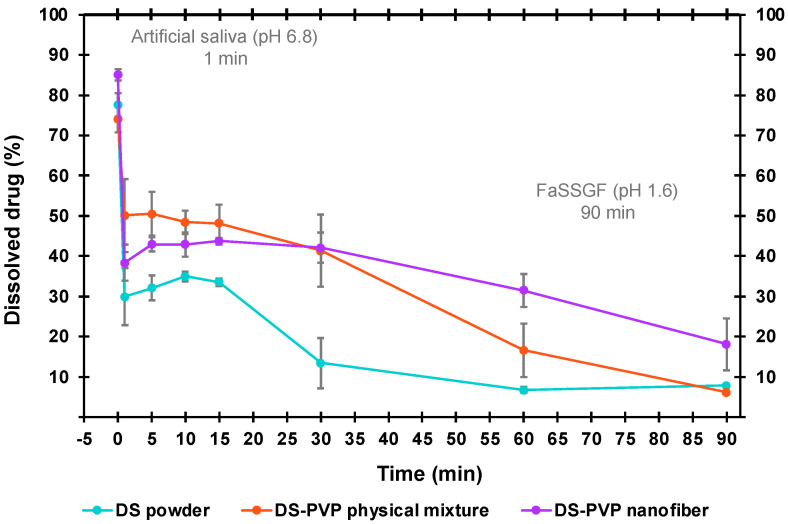
Result of the AS-to-FaSSGF dissolution method in the case of pure diclofenac sodium (DS), DS-PVP physical mixture, and 10 *w*/*w*% DS-PVP nanofiber matrix.

The idea behind the second tested in vitro dissolution method was the swallowing of the drug mixed with saliva. This way, the DS could reach the stomach and interact with the gastric fluid. Since this aspect has not been widely studied, a novel in vitro drug release method, called “AS-to-FaSSGF”, was developed to simulate the natural conditions of drug transit and release (Figure 10).

In the first minute of the “AS-to-FaSSGF” method, the dose was placed into 5 mL of pH 6.8 AS. The DS, due to its salt form, dissolved well in this medium. The dissolved drug amount was 85 ± 1%, 74 ± 3%, and 78 ± 3% in the case of the NF, physical mixture, and pure DS powder, respectively. The NF showed significantly better dissolution in AS in one minute.

After exactly one minute, the liquid was poured into 895 mL pH 1.2 FaSSGF. Since the solubility of the DS is reduced by lowering the pH, the drug precipitated in the second phase. Fast precipitation was observed after the pouring, then the concentration constantly decreased until the end of the measurement. Here, 18 ± 6.5%, 8 ± 0.1%, and 6 ± 0.3% of the initial DS remained dissolved in the case of the NF, physical mixture, and pure DS powder, respectively. The precipitation was visible to the bare eye.

Although the trend was the same for all three samples, there were significant differences between the curves. The DS released from the NF matrix was significantly higher (*p* < 0.05) than the pure drug between 5 and 90 min and the physical mixture at 60 and 90 min. The presence of the PVP improved the solubility, since the physical mixture showed significantly higher (*p* < 0.05) dissolution than the DS powder between 1 and 30 min.

Overall, the “AS-to-FaSSGF” in vitro dissolution method underlined the relevance of an orodispersible dosage form when formulating the DS, as the solubility decreased drastically in simulated gastric fluid.

### 3.7. Biocompatibility of the Nanofibers

**Figure 11:**
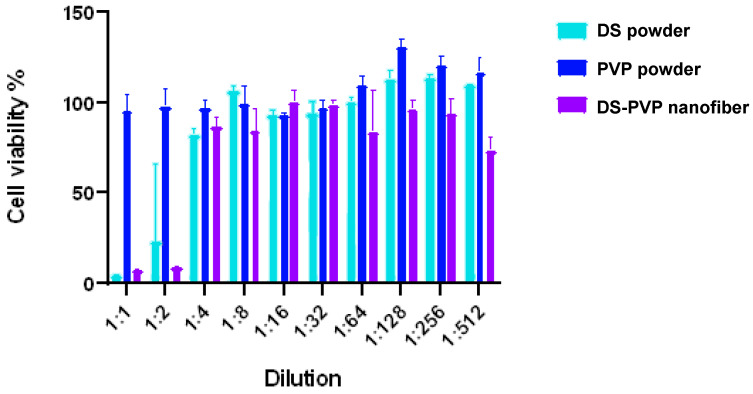
Result of the MTT test comparing the biocompatibility of the pure diclofenac sodium (DS), pure PVP, and the DS-PVP nanofiber matrix.

The biocompatibility of the double-needle electrospun sample and the two pure powders was tested using an MTT assay on Caco-2 cells (Figure 11). According to the results, all three samples were cytocompatible up to 0.25 mg/mL (1:4 dilution). However, in higher concentrations, the DS was cytotoxic both in pure form and in the NF. The pure PVP was cytocompatible even at a 1 mg/mL concentration.

### 3.8. Storage Stability of the Diclofenac-Loaded Nanofibers

**Figure 12:**
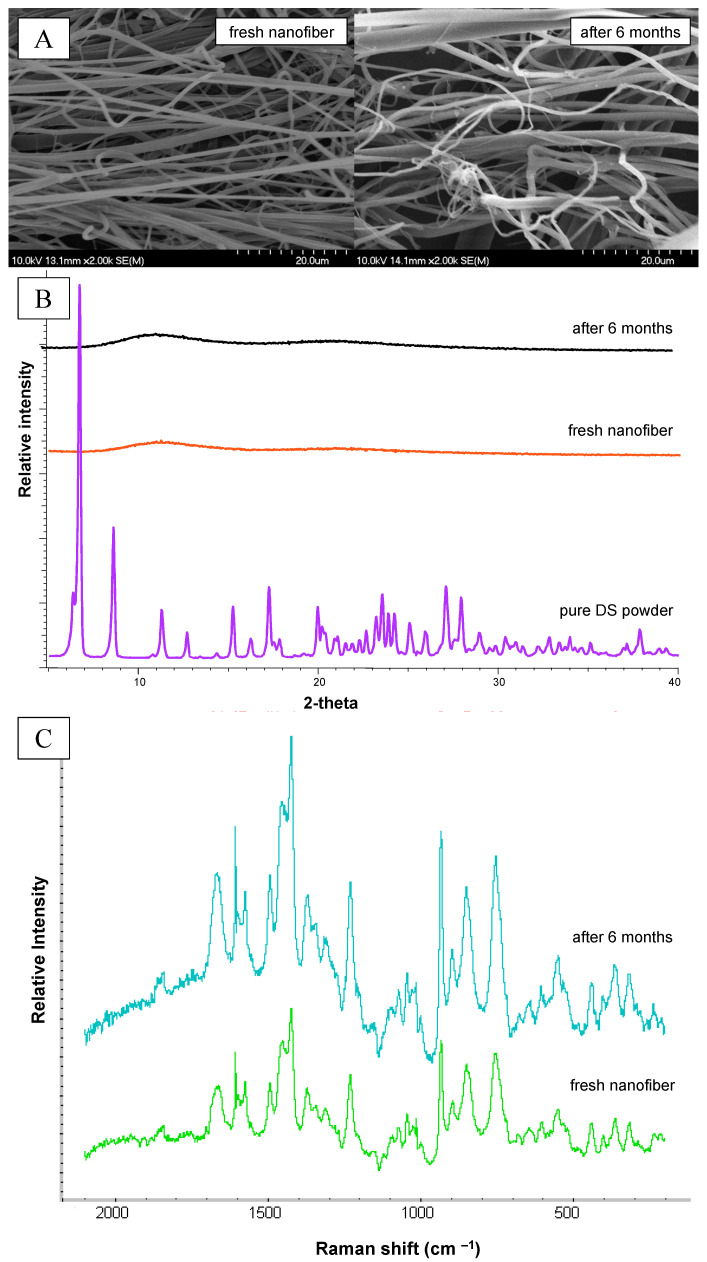
SEM images (**A**), XRPD diffractogram (**B**), and Raman spectra (**C**) of the fresh and the stored double-needle electrospun nanofiber at the beginning and end of the 6-month stability test.

The storage stability of the NFs prepared by double-needle ES was investigated in three aspects: morphology, crystallinity, and chemical stability (Figure 12). All three methods confirmed that the NF matrix was stable for 6 months. The SEM analyses showed that the morphology was still satisfactory. The autonomy of the fibers was maintained, no fusion occurred, and their surface remained smooth (Figure 12A). The measured average fiber diameter did not differ significantly. The XRPD measurement confirmed that no recrystallization occurred during storage since the diffractogram remained flat without any distinctive peaks (Figure 12B). The Raman spectra of the fresh and the stored fibrous samples were similar, and the fingerprint pattern was maintained (Figure 12C). So, neither chemical degradation, nor any other chemical transformation, occurred. The DS-loaded NF matrix was chemically stable over the 6 months.

## 4. Discussion

The most commonly used equipment for NF production is the needle-based ES. Besides its many advantages, one of its biggest disadvantages is its low productivity. Improving this requires more needles connected side by side. In this way, industrial-scale production is also possible.

In this study, DS-loaded PVP NFs were prepared using a double-needle ES device. The needle holder was designed and 3D-printed. The angle formed by the needle tips was adjustable, ensuring that the NFs on the collector formed a single unified patch instead of two separate ones. The process was suitable for the preparation of PVP NF matrices from a 1,300,000 Mw PVP solution with a concentration between 7.5 and 15 *w*/*w*%.

First, drug-free PVP solutions were electrospun to investigate the relationship between the polymer concentration and the fiber morphology. As the polymer concentration increased, the viscosity of the ES solution, as well as the average fiber diameter, increased. A concentration that was too low caused inappropriate viscosity, which led to electrospraying instead of electrospinning. From the 5 *w*/*w*% PVP solution, almost-spherical polymer nanoparticles were formed by the spraying. As the concentration was increased, the globes elongated first into red-blood-cell-shaped structures, then to proper fibers. Overall, the fiber formation was optimal in the case of 12.5 *w*/*w*% PVP solution with an applied voltage of 15 kV and a pumping rate of 0.5 mL/h.

Secondly, DS-loaded NFs were prepared by double-needle ES using the optimal process parameters. The addition of the drug drastically decreased the average fiber diameter. This was probably due to the changed material properties, since the addition of the DS salt increased the conductivity of the ES solution. Previously, our research group observed a similar result with an ES solution of the same polymer and ciprofloxacin [27].

Afterwards, the physicochemical properties of the DS-PVP NFs were investigated. The results proved that the electrospun formulation was an amorphous solid dispersion, the EE% was more than 99%, and the drug distribution was homogeneous within the NF matrix. These properties, and the general characteristics of NFs, such as high specific surface area, predicted rapid disintegration and immediate dissolution. Moreover, fast disintegration of the water-soluble PVP matrix was expected, further promoting the efficient release and subsequent absorption of the drug. The rapid disintegration was proved by two different methods, namely free immersion and simulated tongue. Both methods confirmed the fast disintegration of the nanofibrous formulation.

In vitro dissolution studies are cornerstones of pharmaceutical research. Orodispersible formulations require immediate release in the oral cavity. The DS-PVP NF matrix met the requirements of the USP, as in AS, the dissolution was complete within 10 min. Additionally, a new “AS-to-FaSSGF” dissolution method was developed to test the natural conditions of swallowing the drug. The method used two dissolution media, a small volume of AS and the generally used 900 mL of FaSSGF. Since the solubility of the DS is pH-dependent, the shift to acidic conditions caused a remarkable decrease in the solubility which led to visible precipitation. The DS amount that remained dissolved was significantly higher in the case of the NFs. Therefore, it can be beneficial to use it as an orodispersible formulation.

Finally, the storage stability of the DS-PVP NF matrices confirmed that the formulation was stable for 6 months. Additionally, the cytocompatibility of the ingredients and the electrospun fibers was proved.

Overall, the developed nanofibrous formulation can be a promising candidate as a novel orally disintegrating dosage form. The most common orally disintegrating dosage forms, which are on the market, are the orally disintegrating tablet (ODT) and orodispersible film (ODF). Generally, tablets are a widely favored dosage form due to their ease of manufacturing with uniform mass and consistent dosing. However, ODTs typically exhibit poor mechanical strength, as achieving rapid disintegration requires a porous structure [28]. Another drawback of ODTs is the need for a high amount of excipients to mask the bitterness of the active pharmaceutical ingredient [29]. A proposed solution to these issues is using flexible ODFs because the polymer matrix can effectively mask unpleasant tastes. However, a significant disadvantage of these films is their considerably slower drug release [29]. Orodispersible nanofiber membranes could be an alternative, as they combine the advantages of both dosage forms; they are more flexible than fragile, and have a porous structure, allowing for extremely rapid disintegration. Their potential drawback lies in their production costs, as nanofiber fabrication is a novel technology in the pharmaceutical industry compared to conventional tableting, and its implementation and regulatory approval may require more resources.

## 5. Conclusions

In conclusion, the preparation of the double-needle electrospun PVP NF was optimized, DS was loaded into the fibers, and the formulation was investigated. In order to achieve an orodisperse dosage form, the salt form of the drug and a water-soluble polymer were selected. Many properties facilitated the rapid disintegration and the fast dissolution, i.e., the large specific surface area, the structure of an amorphous solid dispersion, the good wettability, and the porous structure of the NF matrix. Both the rapid disintegration and the immediate dissolution were proved by two different methods. The formulation was biocompatible and stable for 6 months.

## Figures and Tables

**Table 1:** Viscosity and fiber diameter of the different drug-free formulations prepared by varying the PVP concentration.

Sample Name	PVP Concentration (*w*/*w*%)	Viscosity (mPa·s)	Fiber Diameter (nm)
PVP_5	5	21	N.A.
PVP_7.5	7.5	71	431 ± 143
PVP_10	10	164	886 ± 217
PVP_12.5	12.5	350	1427 ± 216
PVP_15	15	657	1564 ± 267

## Data Availability

No new data were created or analyzed in this study. Data sharing is not applicable to this article.

## Abbreviations

The following abbreviations are used in this manuscript:

ANOVA	analysis of variance
AS	artificial saliva
AS-to-FaSSGF	artificial saliva-to-Fasted State Simulating Gastric Fluid
CCD	charge-coupled device
DL%	drug loading
DS	diclofenac sodium
EE%	entrapment efficiency
ES	electrospinning
FaSSGF	Fasted State Simulating Gastric Fluid
FDA	U.S. Food and Drug Administration
HPMC	hydroxypropyl methylcelluloses
HPLC	High Performance Liquid Chromatography
MEM	Minimal Essential Medium
MTT	thiazolyl blue tetrazolium bromide
NF	nanofiber
ODF	orodispersible film
ODT	orally disintegrating tablet
PBS	phosphate-buffer solution
PEO	polyethylene oxide
PVA	polyvinyl alcohol
PVP	polyvinylpyrrolidone
SEM	scanning electron microscopy
USP	United States Pharmacopeia
UV	ultraviolet
UV-Vis	ultraviolet-visible
XRPD	X-ray powder diffraction
